# Revealing the atmospheres of highly irradiated exoplanets: from ultra-hot Jupiters to rocky worlds

**DOI:** 10.1007/s10509-023-04183-5

**Published:** 2023-03-29

**Authors:** Megan Mansfield

**Affiliations:** Steward Observatory, University of Arizona, Tucson, 85715 AZ USA

**Keywords:** Planets and satellites: atmospheres, Planets and satellites: gaseous planets, Planets and satellites: terrestrial planets

## Abstract

Spectroscopy of transiting exoplanets has revealed a wealth of information about their atmospheric compositions and thermal structures. In particular, studies of highly irradiated exoplanets at temperatures much higher than those found in our solar system have provided detailed information on planetary chemistry and physics because of the high level of precision which can be obtained from such observations. Here we use a variety of techniques to study the atmospheres of highly irradiated transiting exoplanets and address three large, open questions in exoplanet atmosphere spectroscopy. First, we use secondary eclipse and phase curve observations to investigate the thermal structures and heat redistribution of ultra-hot Jupiters, the hottest known exoplanets. We demonstrate how these planets form an unique class of objects influenced by high-temperature chemical effects such as molecular dissociation and H^−^ opacity. Second, we use observations of helium in the upper atmosphere of the exo-Neptune HAT-P-11b to probe atmospheric escape processes. Third, we develop tools to interpret *JWST* observations of highly irradiated exoplanets, including a data analysis pipeline to perform eclipse mapping of hot Jupiters and a method to infer albedos of and detect atmospheres on hot, terrestrial planets. Finally, we discuss remaining open questions in the field of highly irradiated exoplanets and opportunities to advance our understanding of these unique bodies in the coming years.

## Introduction

The main goals of exoplanet atmosphere spectroscopy are to determine exoplanets’ compositions and thermal structures in order to further our understanding of planetary formation, physics, and chemistry. The study of extrasolar planets offers an opportunity to investigate planetary origins and climate in a broader context and across a much wider population of planet types than studies of our solar system. In particular, spectroscopic observations of transiting planets can reveal information on their atmospheric compositions and thermal structures. In this review, we present observations of highly irradiated exoplanets aimed at addressing three large, open questions in exoplanet atmosphere spectroscopy. First, what are the primary processes impacting the thermal structures of ultra-hot Jupiters, gas giant planets with equilibrium temperatures above 2000 K, and how do those processes affect their observed emission spectra and phase curves? Second, how does atmospheric escape sculpt the population of hot exoplanets? And third, how can we use the new capabilities of *JWST* to further advance our understanding of highly irradiated exoplanets?

In Sect. 2 we present a series of observations which reveal the thermal structures and heat transport of ultra-hot Jupiters. We present *Hubble Space Telescope* (*HST*) emission observations of the ultra-hot Jupiter HAT-P-7b, which along with other studies led to the realization that ultra-hot Jupiter spectra are impacted by molecular dissociation (Mansfield et al. 2018; Arcangeli et al. 2018; Kreidberg et al. 2018; Parmentier et al. 2018). We next present a *Spitzer Space Telescope* phase curve of KELT-9b, the hottest known exoplanet, which shows enhanced energy transport due to dissociation (Mansfield et al. 2020). We then expand to a broad study of high-temperature chemistry through a population study of all *HST* hot Jupiter emission spectra (Mansfield et al. 2021, 2022). In Sect. 3, we present a discovery of helium escape in the *HST* transmission spectrum of the exo-Neptune HAT-P-11b (Mansfield et al. 2018). In Sect. 4, we present modeling tools to interpret future *JWST* observations of highly irradiated exoplanets. First, we present a data analysis pipeline that can be used to interpret *JWST* eclipse mapping observations of hot Jupiters, which will produce 3D maps of these planets’ daysides (Mansfield et al. 2020). We then present a model of inferred albedos for hot, terrestrial planets which can be used to determine whether such planets have atmospheres, a prerequisite for habitability (Mansfield et al. 2019). Finally, we conclude in Sect. 5.

## Revealing the atmospheres of ultra-hot Jupiters

### An *HST*/WFC3 thermal emission spectrum of HAT-P-7b

**Fig. 1:**
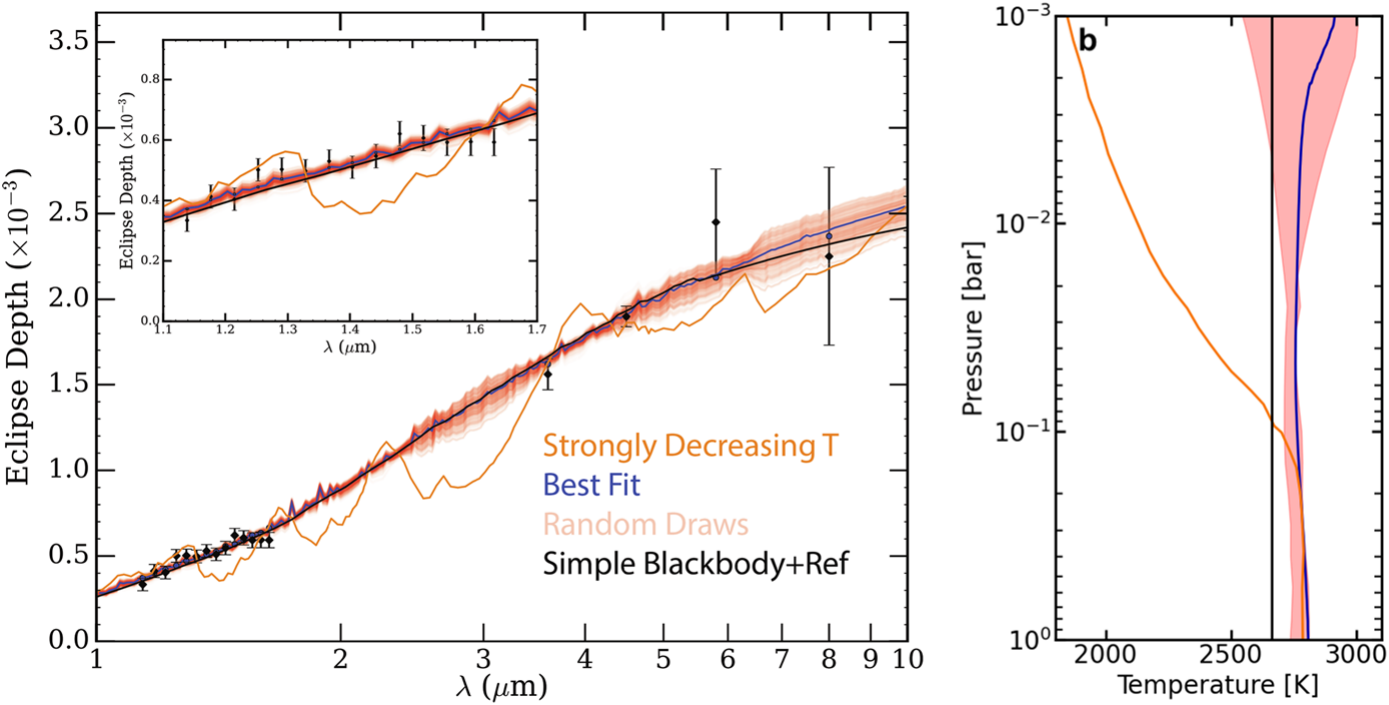
**(a)** Secondary eclipse spectrum of HAT-P-7b compared to a suite of theoretical models. Black points with $1\sigma $ error bars represent observations with *HST*/WFC3 (Mansfield et al. 2018) and *Spitzer* (Wong et al. 2016). The inset zooms in on the WFC3 data. The dark blue line represents the best-fitting 1D self-consistent model (Arcangeli et al. 2018; Mansfield et al. 2021), and the surrounding red lines show 500 spectra randomly drawn from the posterior. Blue points outlined in black show the best-fitting 1D model binned to the data resolution. The black line shows a best-fit blackbody. The orange line shows the expected emission spectrum for a model with a monotonically decreasing T-P profile, calculated using the methods of Fortney et al. (2008). **(b)** Corresponding T-P profiles for each model, with the red shaded area showing $1\sigma $ error bars on the best-fit model. The data are consistent with a blackbody and strongly reject the monotonically decreasing model. Figure from Mansfield et al. (2018)

Ultra-hot Jupiters, which have dayside temperatures above 2000 K (Parmentier et al. 2018) are optimal targets for thermal emission observations in secondary eclipse because of their large radii and high temperatures. The first theoretical studies of hot Jupiters predicted that those with temperatures below $\approx 2000$ K would have non-inverted temperature-pressure (T-P) profiles and thus show absorption features in their secondary eclipse spectra, while those at higher temperatures would have inverted T-P profiles and emission features (Fortney et al. 2008; Hubeny et al. 2003). In order to investigate these predictions, we observed a secondary eclipse of the hot Jupiter HAT-P-7b, which has a dayside temperature of $\approx 2600$ K, with the *HST* Wide Field Camera 3 (WFC3) instrument between $1.1-1.7$ μm (Mansfield et al. 2018). The secondary eclipse spectrum is shown in Fig. 1 compared to several models. We found that the spectrum is blackbody-like and clearly rejects a monotonically decreasing T-P profile.

We compared the data to both 3D general circulation models (GCMs) and 1D self-consistent thermochemical equilibrium models (Arcangeli et al. 2018; Mansfield et al. 2021). Both models preferred an inverted T-P profile, but in both cases the spectrum did not show the previously predicted water emission features because of water dissociation. In both models, the upper atmosphere was heated to high enough temperatures that water began to dissociate. Water dissociation becomes important to shaping ultra-hot Jupiter thermal emission spectra at temperatures above $\approx 2200$ K (Parmentier et al. 2018; Mansfield et al. 2021), which from Fig. 1 is below the dayside temperature of HAT-P-7b. The dissociation limited the range of pressures which could be probed in the WFC3 bandpass, which is primarily sensitive to water opacity. Therefore, the observations were restricted to probing a relatively small, nearly isothermal range of pressures, resulting in the blackbody-like spectrum. The 1D modeling additionally showed that HAT-P-7b has relatively inefficient heat redistribution. Based on our model assumptions, we measured a sub-stellar metallicity for HAT-P-7b ([M/H] = $-0.87^{+0.38}_{-0.34}$) and placed an upper limit on the carbon-to-oxygen ratio (C/O < 1 at 99% confidence).

This study was one of several *HST* observations which found blackbody-like spectra for ultra-hot Jupiters (Arcangeli et al. 2018; Kreidberg et al. 2018; Parmentier et al. 2018). In all of these studies, it was found that water dissociation similarly limited the pressures probed by eclipse spectroscopy. In addition, studies of planets slightly hotter than HAT-P-7b showed that H^−^ opacity could also act to limit the visibility of water emission features (Arcangeli et al. 2018; Kreidberg et al. 2018; Parmentier et al. 2018; Lothringer et al. 2018). These results combined demonstrated the unique influence of high-temperature chemistry on the emission spectra and thermal structures of ultra-hot Jupiters.

### Hydrogen dissociation in the atmosphere of KELT-9b

As demonstrated in Sect. 2.1, molecular dissociation can have a significant impact on the thermal structures of ultra-hot Jupiters (Parmentier et al. 2018; Lothringer et al. 2018). In addition to the dissociation of water, molecular H_2_ was also predicted to dissociate into atomic hydrogen on the daysides of ultra-hot Jupiters and recombine into H_2_ on their nightsides (Bell and Cowan 2018; Komacek and Tan 2018; Parmentier and Crossfield 2018). This process was predicted to distribute significant energy, with heat deposited in the regions where H recombines into H_2_ (Bell and Cowan 2018; Komacek and Tan 2018).

**Fig. 2:**
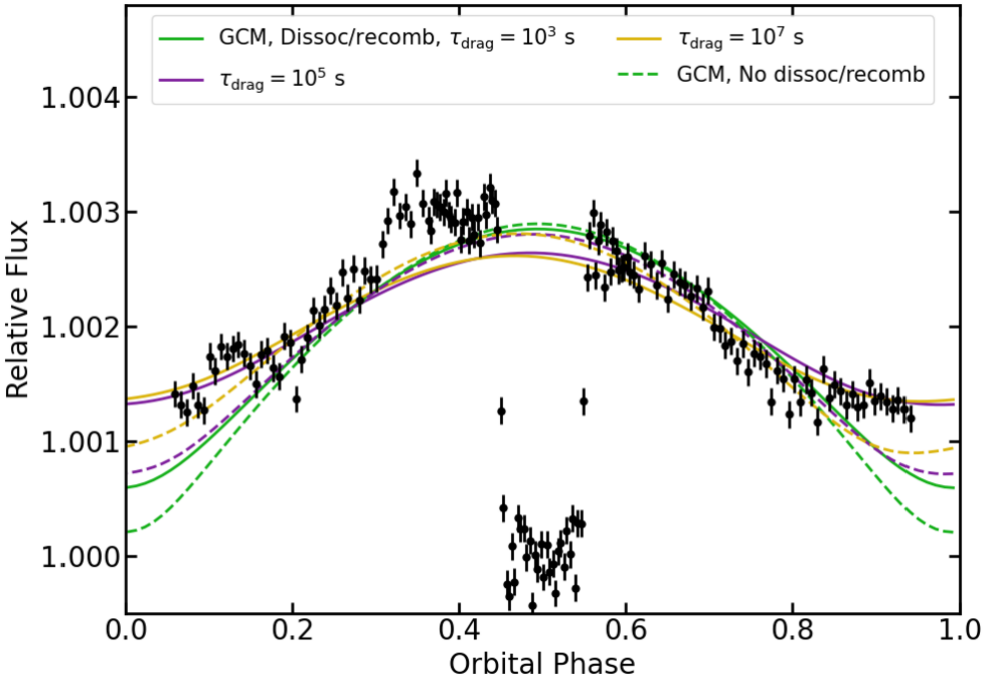
Phase-folded phase curve of KELT-9b (black data points, Mansfield et al. 2020). The transit at phases of 0 and 1 is omitted to better show the phase variation. Green, purple, and gold lines show GCMs with drag timescales of $10^{3}$, $10^{5}$, and $10^{7}$ seconds, respectively (Tan and Komacek 2019). Solid and dashed lines indicate GCMs with and without the effects of H_2_ dissociation and recombination, respectively. Figure adapted from Mansfield et al. (2020)

In order to test this prediction, we observed a phase curve of KELT-9b with the *Spitzer Space Telescope* at 4.5 μm (Mansfield et al. 2020). KELT-9b is the hottest known exoplanet, with a dayside temperature of $\approx 4500$ K (Gaudi et al. 2017). The resulting phase curve is compared to a set of GCMs (Tan and Komacek 2019) in Fig. 2. The phase curve showed a day-night temperature contrast of

1 $$ A_{T}=\frac{T_{day}-T_{night}}{T_{day}}=0.440^{+0.017}_{-0.016} $$

and a phase offset of $18.7^{+2.1}_{-2.3}$°. We found that the relatively low day-night contrast was best matched by GCMs including the effects of H_2_ dissociation and recombination and weak drag, while those without H_2_ dissociation/recombination and/or with strong drag predicted too-large amplitudes and too-cold nightsides. H_2_ dissociation and recombination are necessary to explain the low day-night contrast because KELT-9b has an extremely short radiative timescale ($\tau _{rad}\approx 30$ s) compared to its advective timescale ($\tau _{adv}\approx 4\times 10^{4}$ s), so without the effect of H_2_ dissociation/recombination models predict a very high day-night contrast of $A_{T}=0.999$ (Showman and Guillot 2002; Komacek and Showman 2016).

Although the GCMs including H_2_ dissociation/recombination were able to provide an explanation for the low observed phase curve amplitude, none of the GCMs could explain the large phase offset. All of the GCMs predicted a phase offset of no more than 5°, which is inconsistent with our observations at $>5\sigma $ confidence. One possible explanation for the large observed phase offset is magnetohydrodynamic effects not currently included in the GCM used in this work. Another possibility is that the phase offset is due to variability, as our relatively low-resolution GCMs limit the ability to measure time variability (Cho et al. 2003; Skinner and Cho 2021; Menou 2020). We note that the low resolution of our GCMs should not impact the other main results of this work, as high resolution is not required to capture the main elements of hot Jupiter atmospheric circulation (Menou 2020). However, the level of variability in hot Jupiter atmospheres has yet to be observationally constrained, so the impact of this variability on phase curves is an ongoing focus of exoplanet studies. Future work investigating hot Jupiter variability with *JWST* and future theoretical studies into how magnetic effects influence both the phase curve offset and amplitude (e.g., Rogers and Komacek 2014; Rogers 2017; Hindle et al. 2019) could shed light on the remaining discrepancies between the Spitzer observations and GCMs.

### A survey of hot Jupiter thermal emission spectra with evidence for compositional diversity

Sections 2.1 and 2.2 demonstrate how the population of ultra-hot Jupiters are affected by molecular dissociation in their atmospheres. The combined influence of molecular dissociation and H^−^ opacity provide good explanations for several featureless *HST*/WFC3 secondary eclipse spectra of ultra-hot Jupiters (e.g., WASP-18b, Arcangeli et al. (2018); WASP-103b, Kreidberg et al. (2018)). However, the population of ultra-hot Jupiters show a diversity of spectra (Changeat et al. 2022), including those with emission features (e.g., WASP-121b, Mikal-Evans et al. 2020) and absorption features (e.g., Kepler-13Ab, Beatty et al. 2017) which cannot be explained by molecular dissociation and H^−^ opacity alone.

In order to better understand this diversity of observed hot Jupiter secondary eclipse spectra, we conducted a population study of all 20 hot Jupiters which have been observed in secondary eclipse with *HST*/WFC3 between $1.1-1.7$ μm (Mansfield et al. 2021, 2022). The primary molecular opacity source in hot Jupiter atmospheres in this wavelength range is water, so these observations primarily probe the visitility of water absorption in the planetary spectrum. We reduced and analyzed secondary eclipse spectra for seven new planets and combined these new spectra with one reanalysis of a published spectrum and 12 results from the literature (Wilkins et al. 2014; Mansfield et al. 2018; Nikolov et al. 2018; Crouzet et al. 2014; Line et al. 2016; Ranjan et al. 2014; Stevenson et al. 2014; Arcangeli et al. 2018; Haynes et al. 2015; Kreidberg et al. 2014, 2018) to form a complete sample of all secondary eclipse spectra observed in this wavelength range. For each observed spectrum, we quantified the strength of observed water absorption or emission with the water feature strength metric defined in Mansfield et al. (2021).

We also constructed a grid of self-consistent, radiative-convective-thermochemical equilibrium 1D models to compare to the observed hot Jupiter spectra (Arcangeli et al. 2018; Mansfield et al. 2021). These models were created for a range of planetary equilibrium temperatures and parameterized with a set of five parameters: the stellar effective temperature ($T_{eff}$), the planetary gravity ($g$), the planetary metallicity ($\left [ \frac{\mathrm{M}}{\mathrm{H}}\right ]$), the planetary carbon-to-oxygen ratio ($\frac{\mathrm{C}}{\mathrm{O}}$), and the planetary internal temperature ($T_{int}$). We explored a range of values for all parameters in order to investigate the relative impact of changing different parameters on the resulting water feature strength.

**Fig. 3:**
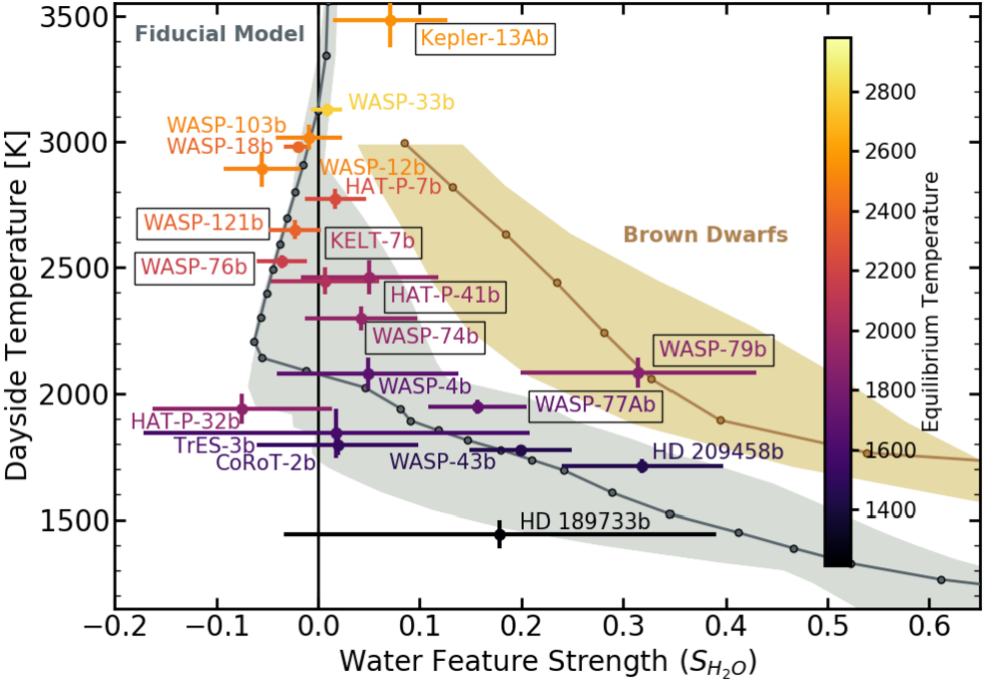
*HST* water feature strength diagram comparing observed secondary eclipse spectra to 1D self-consistent model predictions. The y-axis shows the observed dayside temperature, and the x-axis shows the water feature strength ($S_{H_{2}O}$), both as defined in Mansfield et al. (2021). Featureless, blackbody-like spectra have $S_{H_{2}O}=0$ and absorption/emission features have positive/negative values of $S_{H_{2}O}$, respectively. The gray line and points show the fiducial hot Jupiter models, which use system parameters for a standard hot Jupiter (stellar effective temperature $T_{eff}=5300$ K, planetary gravity $g=10$ m/s^2^, planetary metallicity $\left [\frac{\mathrm{M}}{\mathrm{H}}\right ]=0.0$, planetary carbon-to-oxygen ratio $\frac{\mathrm{C}}{\mathrm{O}}=0.55$, and planetary internal temperature $T_{int}=150$ K). The light gray shaded region shows the full range of hot Jupiter model predictions assuming a range of values for these parameters. Similarly, the brown line and points show fiducial brown dwarf models ($g=1000$ m/s^2^, $\left [\frac{\mathrm{M}}{\mathrm{H}}\right ]=0.0$, $\frac{\mathrm{C}}{\mathrm{O}}=0.55$), and the tan shaded region shows the full range of brown dwarf models assuming different values for $g$ and $\left [\frac{\mathrm{M}}{\mathrm{H}}\right ]$. Colored points with $1\sigma $ error bars show all planets with observed *HST*/WFC3 spectra, and boxes around planet names indicate new data reductions from Mansfield et al. (2021) and Mansfield et al. (2022). The color scale indicates the planetary equilibrium temperature. The error bars include uncertainties in the stellar effective temperature. Figure adapted from Mansfield et al. (2021)

Figure 3 shows the water feature strengths for each hot Jupiter observed with *HST*/WFC3 compared to those of the hot Jupiter model grid. The observed water feature strengths generally fall within the range predicted by the hot Jupiter model grid. The models considered here assume elemental abundance ratios that fall within the range of commonly expected outcomes of planet formation models (Mordasini et al. 2016; Ali-Dib 2017; Madhusudhan et al. 2017; Cridland et al. 2019), and we find that these simple models can explain the observed hot Jupiter population well without having to appeal to less likely outcomes of planet formation (e.g., C/O$>1$, Mordasini et al. 2016; Ali-Dib 2017; Cridland et al. 2019) or exotic chemistry. Figure 3 also shows water feature strengths for a set of brown dwarf models. These models demonstrate how brown dwarfs are distinct from the hot Jupiter population because they show absorption features at all temperatures, due to being primarily heated from their interiors, while hot Jupiters heated externally can show emission features at high temperatures.

While the observed water feature strengths generally agree with the model predictions, Fig. 3 shows that the observed population shows a larger scatter in water feature strengths than predicted by any individual model. This scatter is robust to the selection of different data reductions for individual planets from the literature, including when comparing to the uniformly reduced data set of Changeat et al. (2022). By comparing the scatter in water feature strengths obtained by changing each parameter individually, we found that the best explanation for this scatter is modest differences in metallicity (ranging from $0.03-30\times $ solar) and C/O ratio (ranging from 0.01-0.85). Such variation is expected from planet formation models (Mordasini et al. 2016; Ali-Dib 2017) and has been suggested by a handful of transmission spectra studies. Future observations with *JWST* (e.g., The JWST Transiting Exoplanet Community Early Release Science Team et al. 2022) and high-resolution spectrographs on ground-based telescopes (e.g., Snellen et al. 2010) will be able to more precisely constrain this compositional diversity, as well as detect other molecules which can have a secondary impact on the spectral shape in this wavelength region.

## Atmospheric escape from the highly irradiated exoplanet HAT-P-11b

Close-in planets are expected to experience atmospheric escape that is driven by the absorption of the copious high energy radiation they receive from their host stars (Lammer et al. 2003; Lecavelier des Etangs et al. 2004). Such photoevaporation is proposed to sculpt the observed population of close-in exoplanets and create a radius gap between two categories of small planets - those with radii smaller than $1.5R_{\oplus}$, which are likely rocky cores stripped of significant primordial atmospheres, and those with radii larger than $2R_{\oplus}$, which retain some hydrogen and helium in their atmospheres (Lopez and Fortney 2013; Owen and Wu 2013, 2017; Fulton and Petigura 2018; Van Eylen et al. 2018).

Recently, observation of atmospheric absorption in the helium triplet at 10,833 Å has emerged as an effective probe of atmospheric escape from hot Jupiters with ground-based telescopes, *HST*, and *JWST* (Seager and Sasselov 2000; Oklopčić and Hirata 2018; Spake et al. 2018; Allart et al. 2018; Fu et al. 2022). We observed the hot Neptune HAT-P-11b with *HST*/WFC3 between $0.8-1.15$ μm and found the signature of helium absorption in its upper atmosphere (Mansfield et al. 2018). These observations yielded the second detection of helium escaping from a planet with *HST*, and the first time the same signature of atmospheric escape was observed from both ground-based (Allart et al. 2018) and space-based (Mansfield et al. 2018) facilities.

**Fig. 4:**
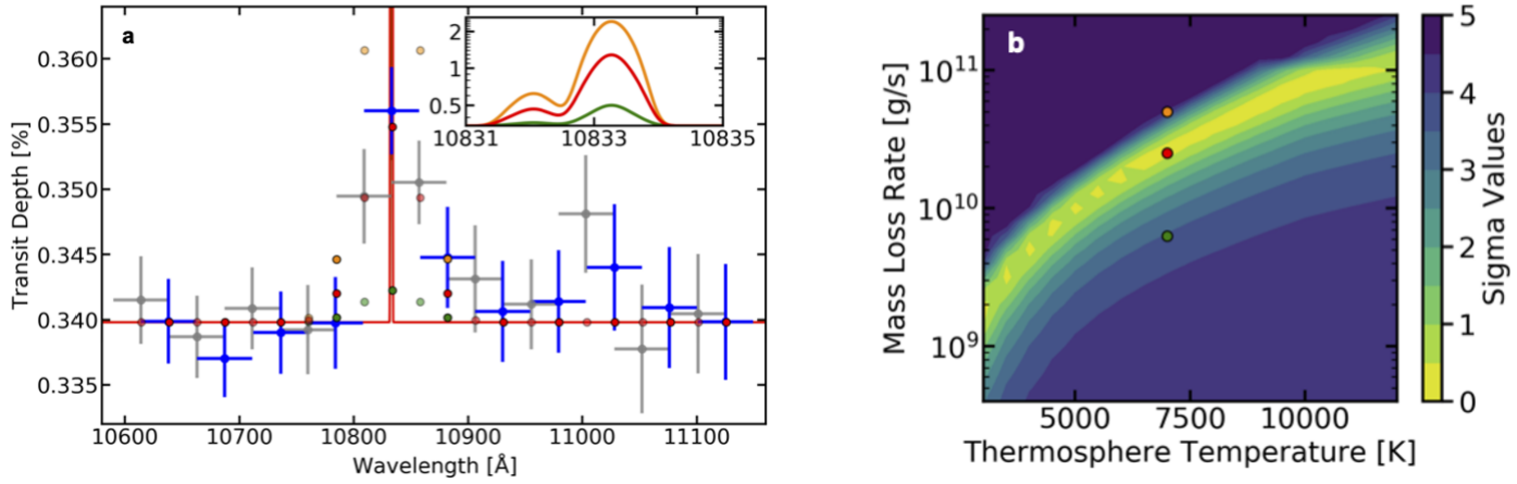
**(a)** Narrowband spectrum of HAT-P-11b (blue and gray points indicating 2-pixel-wide bins with $1\sigma $ error bars) compared to three 1D models of hydrodynamic escape. The red line shows a model with a thermospheric temperature $T_{therm}=7000$ K and a mass loss rate of $\dot{M}=2.5\times 10^{10}$ g/s, which provides an excellent match to the data. For comparison, the green and orange lines show models with $T_{therm}=7000$ K and $\dot{M}=6.3\times 10^{9}$ g/s and $5.0\times 10^{10}$ g/s, respectively. These models are inconsistent with the data at $\geq 3\sigma $ confidence. Blue points show non-overlapping bins. Red, green, and orange points show the models convolved with the G102 instrument resolution (Kuntschner et al. 2009) and binned to the sampling of the data. The inset shows the models at high resolution. **(b)** Contour plot showing the statistical significance of the deviation of the 1D grid models from a good fit to our observations, as a function of thermospheric temperature and mass loss rate. The red, green, and orange points outlined in black show the locations in parameter space of the three models in panel **(a)**. Figures from Mansfield et al. (2018)

We compared the observed helium feature to a grid of models of hydrodynamic escape computed using the methods of Oklopčić and Hirata (2018). These models were parameterized with two values: the thermospheric temperature ($T_{therm}$) and the total mass loss rate ($\dot{M}$). Figure 4 shows the narrowband spectrum of HAT-P-11b around the helium absorption feature compared to three selected models, as well as a contour plot of the fit quality for comparing the full grid of models to the data. Figure 4 shows how the degeneracy between $T_{therm}$ and $\dot{M}$ prevents exact determination of these quantities from our low-resolution observations. However, the range of best fitting parameters cover those previously predicted for HAT-P-11b by hydrodynamic escape simulations (Salz et al. 2016). The range of best fitting models predict a net mass loss of $0.04-2.3$% of the total mass of HAT-P-11b per billion years, which will have a negligible impact on the Neptune-sized planet’s composition. However, future observations of helium loss on smaller planets will help us better understand the evolution of their atmospheres over time.

## Data analysis methods for future *JWST* observations

### The eigenspectra method for spectroscopic eclipse mapping

Secondary eclipses of transiting planets offer valuable opportunities to observe and understand the multidimensional nature of exoplanets (e.g. Williams et al. 2006; Rauscher et al. 2007; Coulombe et al. 2023). As a planet goes behind its star, the stellar limb scans across the dayside hemisphere of the planet, permitting a 2D reconstruction (an eclipse map) of the planetary photosphere by probing the latitudinal structure. The spectral information provided by *JWST* during secondary eclipses will add a third dimension to this mapping, since different wavelengths may probe different altitudes in the planet’s atmosphere (albeit not necessarily through a simple correspondence, Dobbs-Dixon and Cowan 2017; Schlawin et al. 2018). However, eclipse mapping does not provide a perfect proxy for each spatial dimension; there are inherent degeneracies in constructing a spatially-resolved map from secondary eclipse observations, which measure spatially integrated brightness over time. Rauscher et al. (2018) addressed this issue by developing an orthogonal basis of light curves built from linear combinations of spherical harmonic maps, which they term “eigencurves”, to best represent the information available from both phase variations and secondary eclipses at a single wavelength.

**Fig. 5:**
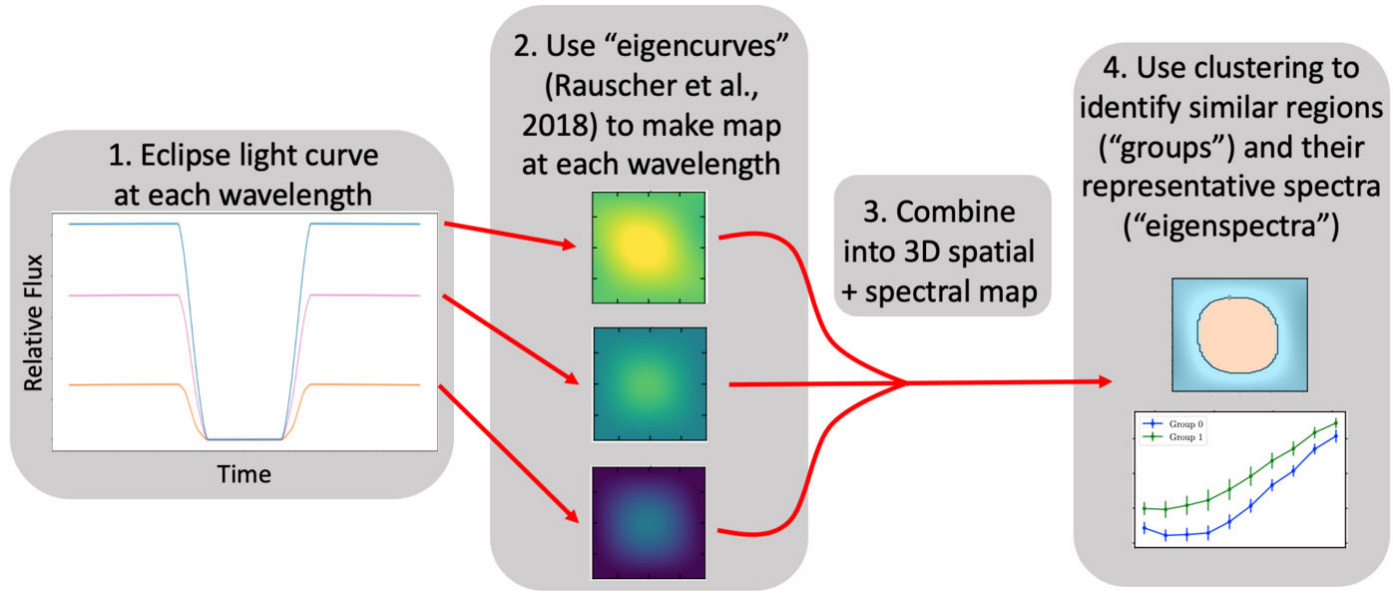
Overview of the eigenspectra method for extracting spatially resolved spectral information from secondary eclipse light curves. We apply the method of Rauscher et al. (2018) and use eigencurves to construct a map separately at each wavelength. We then combine these single-wavelength maps into a 3D spatial + spectral map. We use K-means clustering to identify similar regions on this 3D map (“groups”) and their representative spectra (“eigenspectra”). Figure from Mansfield et al. (2020)

In order to extend this framework to the third dimension of multi-wavelength observations, we developed the “eigenspectra” method (Mansfield et al. 2020). Figure 5 shows an outline of the steps in the eigenspectra method. The method began with systematics-corrected secondary eclipse light curves at each observed wavelength. First, we used the eigencurves method of Rauscher et al. (2018) to produce a map of planetary flux as a function of longitude and latitude at each observed wavelength. These maps are stacked together to create one “three-dimensional” map, where the vertical dimension through the atmosphere is parameterized by wavelength. Finally, we used a K means clustering algorithm (Pedregosa et al. 2011) to identify regions of the map with similar spectral features. Each identified region was termed a “group”, and we extracted a representative spectrum, or “eigenspectrum” from each group by taking the mean of all points included in the group.

We found that this method is effective at identifying broad features across the dayside of a planet, such as large-scale changes in temperature, molecular abundances, or cloud opacity. The maps, which are based upon smoothly-varying spherical harmonics, can not resolve sharp discontinuities such as those caused by storms (Komacek and Showman 2020; Cho et al. 2021) well. However, we tested the eigenspectra method by inputting maps with sharp discontinuities and found that although it may struggle to correctly identify the small spatial extent of a sharp discontinuity, it can readily identify large changes in spectral features, even if those changes appear to happen over a larger spatial scale in the output maps. The eigenspectra method is thus useful for identifying large-scale structure in planet maps, and can additionally be used to predict what scale of features in GCMs will be observable with *JWST*.

Spectroscopic eclipse mapping with *JWST* will advance our understanding of thermal structures and heat redistribution on the daysides of hot Jupiters by revealing spatially-resolved patterns that are inaccessible to phase curves (see Sect. 2.2) or hemisphere-integrated secondary eclipse observations (see Sects. 2.1 and 2.3). For example, spectroscopic eclipse mapping will enable measurements of evolving cloud coverage and molecular dissociation with temperature across the daysides of single objects (e.g., Parmentier and Crossfield 2018; Beatty et al. 2019; Keating et al. 2019; Tan and Komacek 2019; Parmentier et al. 2021). Additionally, measurement of the hotspot offset in both longitude and latitude may reveal the influence of magnetic effects on hot Jupiter circulation (e.g., Batygin and Stanley 2014; Rauscher and Menou 2013; Rogers and Komacek 2014; Beltz et al. 2022). The development of the eigenspectra method represents a step toward a data-driven perspective on the 3D nature of exoplanet atmospheres.

### Identifying atmospheres on rocky exoplanets through inferred high albedo

In addition to enabling spectroscopic eclipse mapping of hot Jupiters, *JWST* will give the first opportunity to look in detail at the atmospheres of small, terrestrial planets, particularly those orbiting M dwarf stars. However, close-in rocky exoplanets orbiting M dwarfs are subjected to intense X-ray and ultraviolet flux (“XUV” flux). This high XUV flux is thought to sculpt the observed population of close-in exoplanets (Lopez and Fortney 2013; Owen and Wu 2013; Rogers 2015; Owen and Wu 2017; Fulton and Petigura 2018; Van Eylen et al. 2018) and may even strip atmospheres entirely off rocky M dwarf exoplanets. Transmission and emission spectra and phase curves can all constrain the presence of an atmosphere on a rocky planet, but in many cases may take many hours of repeated observations to reach a conclusion (e.g., Kreidberg et al. 2014; Morley et al. 2017). We present a method of detecting an atmosphere on a rocky planet through observations of thermal emission, which can be used to infer its albedo at visible wavelengths (Mansfield et al. 2019). Assuming that planetary surfaces are generally low albedo, a high albedo could then be interpreted as evidence of an atmosphere.

**Fig. 6:**
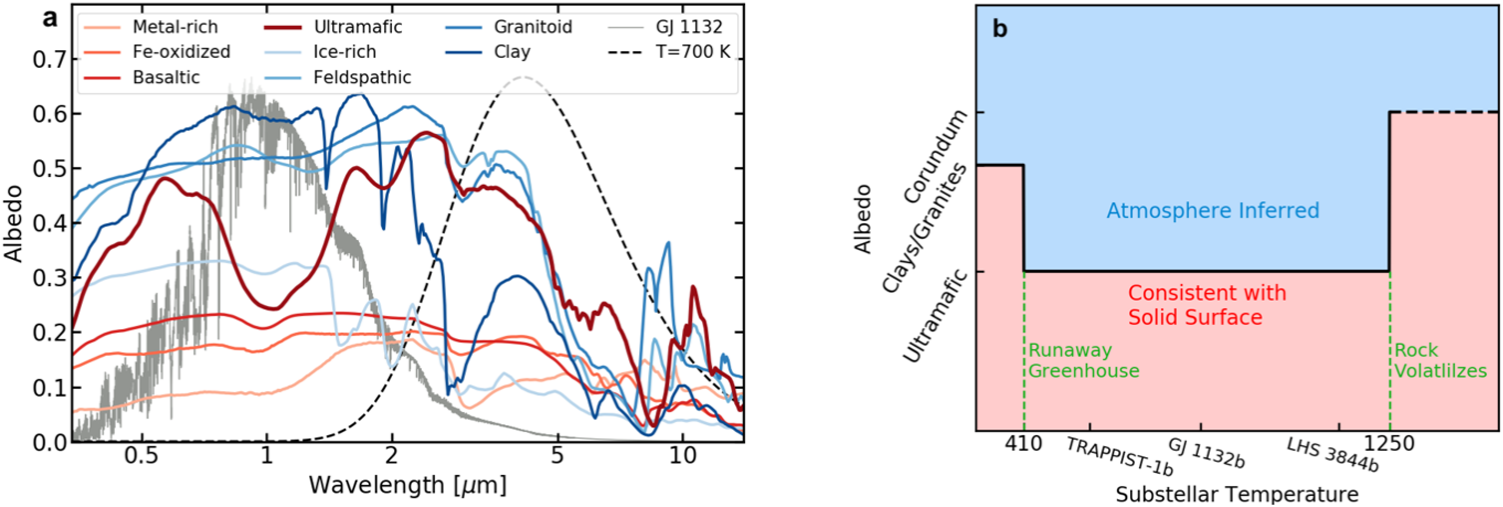
**(a)** Albedo as a function of wavelength for the eight types of planetary surfaces considered here, taken from Hu et al. (2012) (red and blue lines). The solid gray line shows a PHOENIX (Husser et al. 2013) model for the stellar spectrum of GJ 1132, one of the systems modeled here, and the dashed black line shows a blackbody at $T=700$ K, which is the approximate temperature of the dayside of the planet GJ 1132b. Red tinted lines indicate surface compositions that are plausible for planets with substellar temperatures $410< T_{sub}<1250$ K, while blue tinted lines are compositions that are not likely to occur at these temperatures. **(b)** Cartoon demonstrating at what temperatures measurement of a high albedo can unambiguously indicate the presence of an atmosphere. Below a substellar temperature of 410 K, where planets are not guaranteed to have entered a runaway greenhouse, high-albedo, water-rich materials such as clays and granites can form. Above a substellar temperature of 1250 K, the rock partially volatilizes, which may lead to the formation of high-albedo corundum. Between these two extremes, the predicted common rock compositions (shown by red lines in panel (a)) are relatively low albedo, and a measurement of a high albedo could be unambiguously interpreted as an atmosphere (blue region). Labels on the x-axis indicate the substellar temperatures of the three planets considered in detail here, assuming zero albedo and no heat redistribution. Figures from Mansfield et al. (2019)

We investigated the range of planetary temperatures at which all plausible surfaces could reasonably be expected to have low albedos, and thus the presence of an atmosphere could be unambiguously identified. We modeled observations of three representative terrestrial planets (GJ 1132b, TRAPPIST-1b, and LHS 3844b) with surfaces made of eight common terrestrial surface types whose reflectance spectra are shown in Fig. 6 (Hu et al. 2012). We calculated the reflected and emitted light as a function of wavelength for each surface spectrum, using PHOENIX stellar spectra (Husser et al. 2013) to model the stellar irradiation. We used PandExo (Batalha et al. 2017) to simulate observations of the planets with *JWST*/MIRI, then calculated the planetary brightness temperature and albedo inferred from each set of observations.

**Fig. 7:**
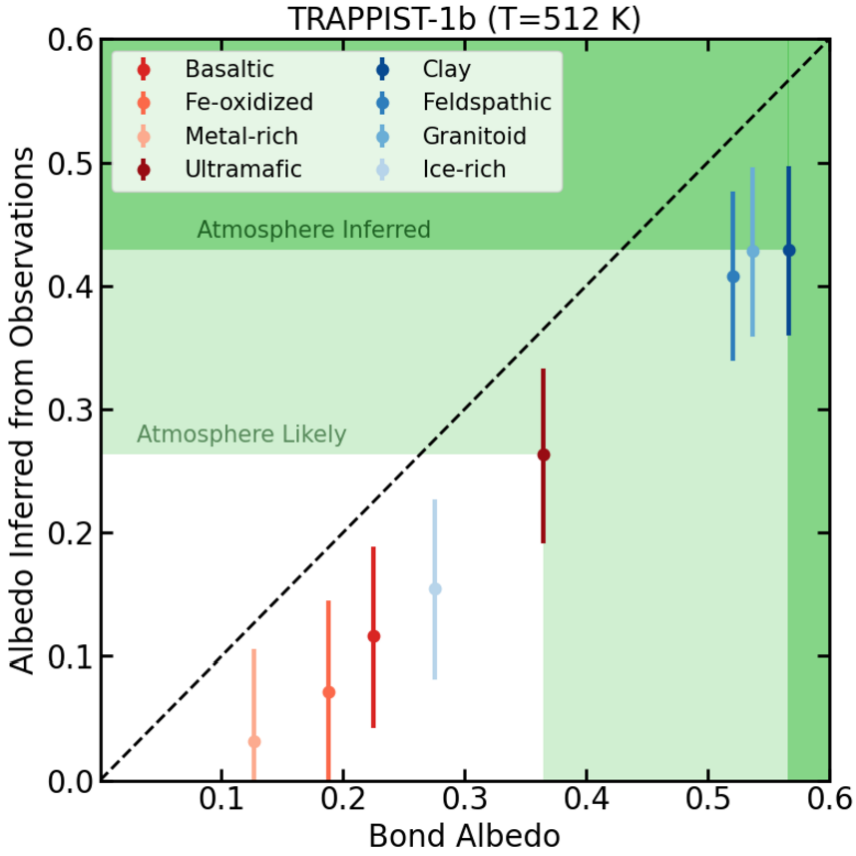
Insolation flux-weighted albedo (Bond albedo) in the wavelength range from 0.1 to 3.5 μm of the eight possible planetary surfaces shown in Fig. 6a compared to the albedo inferred from longer-wavelength observations with *JWST*/MIRI for TRAPPIST-1b. The temperature quoted in the title assumes zero albedo ($\alpha =0$) and no heat redistribution. The error bars indicate $1\sigma $ observational uncertainty for five stacked secondary eclipse observations. The black dashed line shows where the Bond albedo equals the inferred albedo. In all cases, the inferred albedo is lower than the actual albedo because of the difference in albedo as a function of wavelength for each surface type between the optical wavelengths and MIRI/LRS wavelengths. The light green shaded region indicates where the albedo is high enough that an atmosphere is likely, because all surfaces with higher albedos are implausible for planets with $410< T_{sub}<1250$ K. The dark green shaded region indicates where an atmosphere is needed to explain such a high inferred albedo. Figure adapted from Mansfield et al. (2019) with improved inferred albedo calculations based on updates in Whittaker et al. (2022)

Figures 6 and 7 show a summary of our results. We found that four surface types (ice-rich, feldspathic, granitoid, and clay) had high inferred albedos which could potentially be confused with atmospheres. However, a feldspathic crust is only expected to be a major rock-forming component on bodies much smaller than the Earth (Elkins-Tanton et al. 2011; Elkins-Tanton 2012), and the other three of these surface types (ice, granite, and clay) require water, either to form the surface itself or as part of the mineral structure (Campbell and Taylor 1983; Hamano et al. 2013; McSween et al. 2019). Therefore, these surfaces are not expected to exist at temperatures higher than the runaway greenhouse limit (Kopparapu et al. 2013), which we calculated to occur by a substellar temperature of $T_{sub}=410$ K, even when considering the potential cooling effect of clouds (Yang et al. 2013, 2014; Kodama et al. 2018). Additionally, we calculated the rate of partial rock devolatilization at high temperatures (Kite et al. 2016; Fegley and Cameron 1987; Schaefer and Fegley 2009) and found that above substellar temperatures of $T_{sub}=1250$ K, partial devolatilization can produce a high-albedo calcium- and aluminum-rich surface faster than can be destroyed by meteoritic gardening (Warner et al. 2017; Fassett et al. 2017). We thus found that our method of inferred albedo could unambiguously identify the presence of an atmosphere on a planet with a substellar temperature between $410< T_{sub}<1250$ K.

This method of detecting an atmosphere is complementary to that of Koll et al. (2019), who also consider secondary eclipse observations but focus on identifying an atmosphere through heat redistribution. Koll et al. (2019) describe a way to detect atmospheres thicker than 1 bar, while our method allows identification of atmospheres that are too thin to cause significant heat redistribution but still thick enough to host high-albedo clouds. These two methods combined, therefore, allow for detection of atmospheres across a wide range of planets.

## Conclusion

The studies reviewed above use a wide variety of methods to study highly irradiated atmospheres and advance our understanding of planetary formation, chemistry, and physics. One of the main goals of future exoplanet observations will be the characterization of cooler planets more analogous to those in our solar system, in particular of potentially habitable planets. Future ground-based and space-based observatories such as the next generation large UV/optical/IR space telescope (National Academies of Sciences, Engineering, and Medicine 2021), the European Extremely Large Telescope (ELT, Ramsay et al. 2021), the Giant Magellan Telescope (GMT, Fanson et al. 2020), and the Thirty Meter Telescope (TMT, Skidmore et al. 2015) will enable atmospheric characterization at even higher levels of precision. These more precise observations will make it feasible to extend spectroscopic studies from highly irradiated exoplanets down to lower temperatures, which will give us a more complete understanding of the full diversity of known exoplanets.
